# Brain derived neurotrophic factor (BDNF) contributes to the pain hypersensitivity following surgical incision in the rats

**DOI:** 10.1186/1744-8069-4-27

**Published:** 2008-07-17

**Authors:** Chang-Qi Li, Jun-Mei Xu, Dan Liu, Jian-Yi Zhang, Ru-Ping Dai

**Affiliations:** 1 Department of Anatomy and Neurobiology, Xiang-Ya College of Medicine, Central South University, Changsha, PR China; 2 Department of Anesthesia, Xiang-Ya Second Hospital, Central South University, Changsha, PR China

## Abstract

**Background:**

The pathogenic role of brain derived neurotrophic factor (BDNF) in the incisional pain is poorly understood. The present study explores the role of the BDNF in the incision-induced pain hypersensitivity.

**Methods:**

A longitudinal incision was made in one plantar hind paw of isoflurane-anesthetized rats. Dorsal root ganglias (DRG) and spinal cords were removed at various postoperative times (1–72 h). Expression pattern of BDNF was determined by immunohistochemistry and double-labeling immunofluorescence. Lidocaine-induced blockade of sciatic nerve function was used to determine the importance of afferent nerve activity on BDNF expression in the DRG and spinal cord after incision. BDNF antibody was administered intrathecally (IT) or intraperitoneal (IP) to modulate the spinal BDNF or peripheral BDNF after incision.

**Results:**

After hind-paw incision, the BDNF was upregulated in the ipsilateral lumbar DRG and spinal cord whereas thoracic BDNF remained unchanged in response to incision. The upregulated BDNF was mainly expressed in the large-sized neurons in DRG and the neurons and the primary nerve terminals in the spinal cord. Sciatic nerve blockade prevented the increase of BDNF in the DRG and spinal cord. IT injection of BDNF antibody greatly inhibited the mechanical allodynia induced by incision whereas IP administration had only marginal effect.

**Conclusion:**

The present study showed that incision induced the segmental upregulation of BDNF in the DRG and spinal cord through somatic afferent nerve transmission, and the upregulated BDNF contributed to the pain hypersensitivity induced by surgical incision.

## Background

Brain derived neurotrophic factor (BDNF) is a 12.4-kDa basic protein initially isolated from pig brain and widely expressed in the peripheral and central nervous system. In the dorsal root ganglia (DRG), BDNF is expressed in the small- and medium-sized neurons and anterogradely transported to the central terminals of the spinal cord where BDNF is located in large dense-cored vesicles in terminals of primary afferent fibers in groups I and II glomeruli [1,2]. Numerous studies show that BDNF is a modulator of pain in the inflammatory and neuropathic pain. First, in the formalin and carrageenan-induced pain models, BDNF was upregulated in the DRG and spinal cord and sequestering the upregulated BDNF reduced the pain hypersensitivity [3,4]. Second, in another inflammatory pain model induced by complete Freund's adjuvant (CFA) hind paw injection, BDNF was also increased in the spinal cord, and pretreatment with anti-BDNF antiserum abolished the increased number of neurons as well as the pain hypersensitivity after CFA injection [5]. The upregulation of BDNF in the DRG was mediated by nerve growth factor (NGF)-dependent mechanism [6,7]. On the other hand, BDNF is also increased in the medium- and large-sized DRG neurons and their central terminals as well as the activated microglia in spinal cord in the neuropathic pain model in the rat and mouse, and delivery of BDNF antibodies reduced pain-related behavior [8,9]. However, it remains to be ascertained whether BDNF is also involved in another type of pain, post-operative pain.

Post-operative pain, a unique and common form of acute pain, could induce the rest pain and incident pain (a mechanically evoked pain) [10]. Growing evidence demonstrated that non-NMDA receptors were linked to the pain hypersensitivity induced by surgical incision whereas NMDA receptors were believed to play critical roles in other types of pathological pain [11-13]. These reports indicate that there may be distinct neurochemical changes in the spinal cord between incision-induced pain and other types of pathological pain. It is still poorly understood the pathogenic role of BDNF in the DRG and spinal cord in the incision-evoke pain hypersensitivity. In this regard, a previous post-operative study showed that another neurotrophic factor, NGF contributed to the incision-evoked guarding pain behavior [14]. Given that the increased NGF in response to peripheral inflammation could induce the upregulation of BDNF in the DRG and spinal cord, which subsequently phosphorylated the downstream signals including NMDA receptor subunit NR1, tropomyosine receptor kinase B (TrkB) and extracellular regulating kinase (ERK) [15,16], it is possible that BDNF is also associated with the incision-evoked pain hypersensitivity. Supporting this hypothesis, our recent study showed that surgical incision also activated glial cells and upregulated interleukin-1 beta (IL-1β), a proinflammatory cytokine regulated by ERK [17]. Thus, it is rational to postulate that BDNF is associated with the incision-evoked pain hypersensitivity. The present study is thus aimed to investigate the role of BDNF in the pain hypersensitivity induced by incision.

## Results

### Segmental upregulation of BDNF in the spinal cord after hind-paw incision

**Figure 1:**
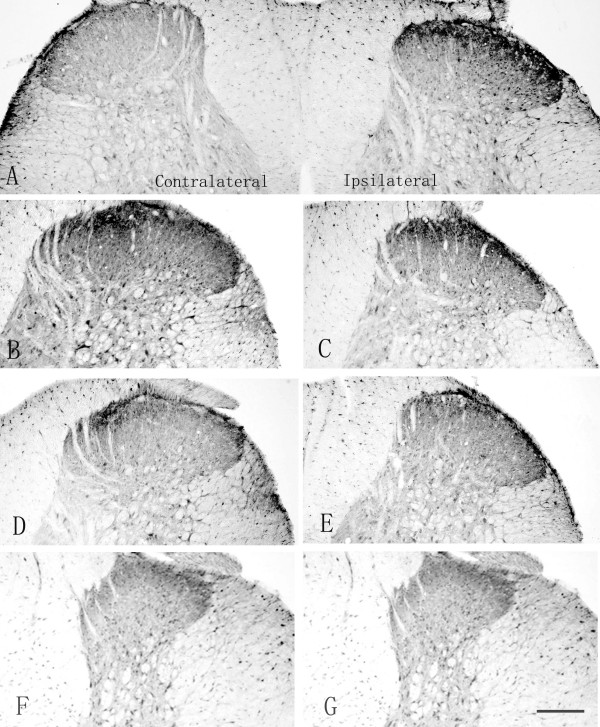
**Segmental upregulation of spinal BDNF after hind-paw incision**. Representative photomicrographs of BDNF-IR density in the lumbar (L4–L5) (Figure 1A-E) and thoracic segments (T1–T2) (Figure 1F and G) of the spinal cord in the rats after hind-paw incision. In the lumbar segments, the increased BDNF expression in the dorsal horns of the ipsilateral spinal cord was observed at 1 hour (Figure 1A), 6 hours (Figure 1B) and 1 day (Figure 1C) after incision. At 3 days after incision, BDNF expression (Figure 1D) was comparable to the sham-operated control (Figure 1E). No significant difference of BDNF expression in thoracic segments between the control (Figure 1F) and 6 hour after incision (Figure 1G). Bar = 150 μm.

At 1 hour after hind-paw surgical incision, the expression of BDNF was increased dramatically in the ipsilateral dorsal horn of the lumbar spinal cord as compared with that in the contralateral side (p < 0.05, Figure 1A and Table 1). Increased BDNF level reached the peak level at 6 hours after incision when the BDNF protein level was about the 2.5 folds of that in contralateral side (p < 0.05, Figure 1B and Table 1). The elevated BDNF-IR positive staining, which appeared to be the axon fibers and neurons, were found in the superficial dorsal horn (lamina I and II). In the deep dorsal horn (lamina III and IV), considerable increased intensity of BDNF-IR was also observed in the neurons (Table 1). Mild upregulation of BDNF was observed at 1 day following surgery (p < 0.05, Figure 1C, Table 1). At 3 days after incision, the expression of BDNF (Figure 1D) was comparable with the sham-operated rats (Figure 1E) (Table 1).

**Table 1:** The OD of BDNF-IR in the spinal cord dorsal horns of lumbar (L4–L5) and thoracic (T1–T2)

OD of BDNF-IR		L4–L5 Spinal Cord					T1–T2 Spinal Cord	
		
		Sham	1h	6h	1d	3d	Sham	6h
Lamina I-II	Contra	0.19 ± 0.03	0.23 ± 0.04	0.22 ± 0.05	0.25 ± 0.04	0.21 ± 0.03	0.18 ± 0.05	0.20 ± 0.04
	Ipsi	0.20 ± 0.04	0.43 ± 0.06*^#^	0.58 ± 0.04*^#^	0.46 ± 0.05*^#^	0.20 ± 0.04	0.20 ± 0.03	0.24 ± 0.05
Lamina III-IV	Contra	0.12 ± 0.07	0.15 ± 0.02	0.16 ± 0.09	0.14 ± 0.03	0.13 ± 0.03	0.17 ± 0.04	0.16 ± 0.03
	Ipsi	0.11 ± 0.04	0.35 ± 0.01*^#^	0.40 ± 0.04*^#^	0.37 ± 0.02*^#^	0.15 ± 0.05	0.14 ± 0.06	0.18 ± 0.01

segments after hind-paw incision

* p < 0.05 vs contralateral side, ^#^p < 0.05 vs sham-operated group

To further explore whether incision-induced BDNF upregulation is mediated by a specific effect in the related segments or unspecific effect in the whole spinal cord, the expression pattern of the BDNF in the thoracic segments was examined. There was no significant alteration for BDNF-IR in the thoracic segments after surgical incision as compared with the sham-operation (Figure 1F and 1G) (Table 1) suggesting that the incision induced segmental BDNF upregulation in the spinal cord.

### Localization of BDNF in the spinal cord following surgical incision

**Figure 2:**
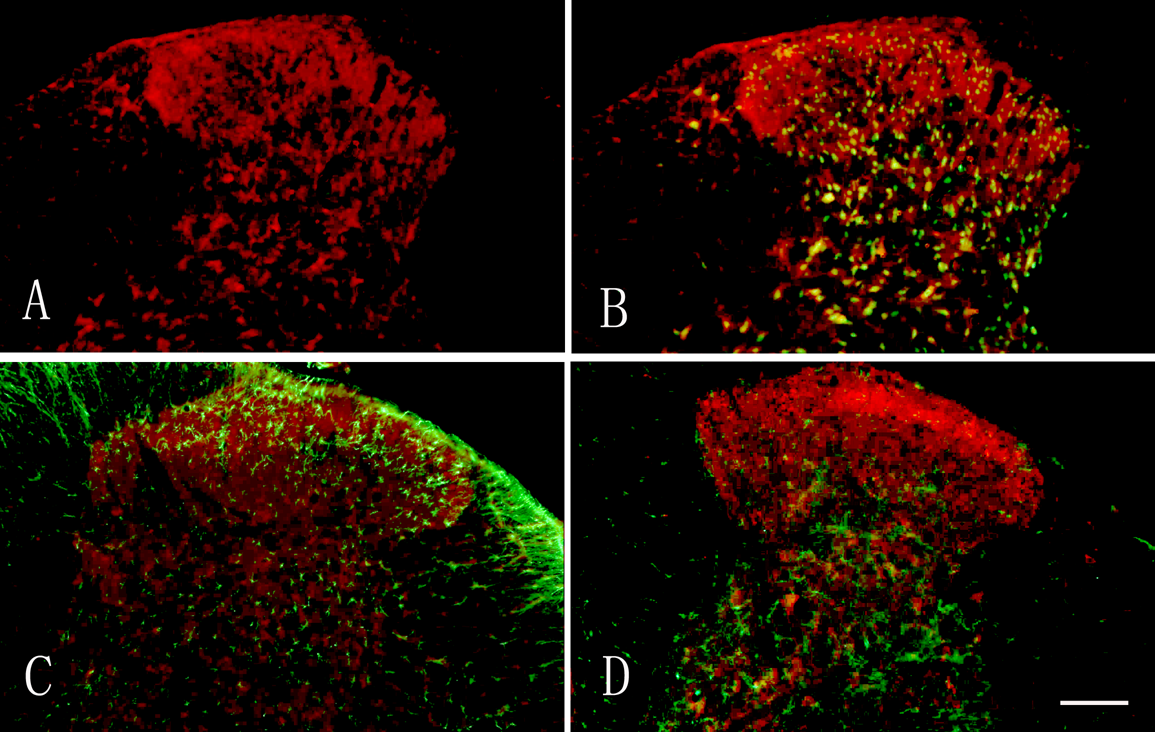
**Localization of BDNF in the spinal cord after hind-paw incision**. Representative photomicrographs of the double immunofluorescence of BDNF (red, Figure 2A) with NeuN-IR (marker of neuron, green in Figure 2B), GFAP-IR (marker of astrocyte, green in Figure 2C) or OX-42 (marker of microglia, green in Figure 2D). Note that abundant BDNF expressing cells were also expressed in the neurons but scarcely expressed in the astrocytes or microglia. Bar = 150 μm.

In attempt to confirm the localization of the upregulated BDNF, double immunofluorescence was performed. As shown in Figure 2, the upregulated BDNF was mainly localized in the neurons and the primary nerve terminals in the superficial layers and the deep layers of the dorsal horns (Figure 2B). Scarce BDNF was expressed in the GFAP-IR cells (Figure 2C), or OX-42-IR cells (Figure 2D). These data suggest that the neurons and nerve terminals be the mainly source of the upregulated BDNF.

### Increased BDNF expression in the DRG after hind-paw incision

**Figure 3:**
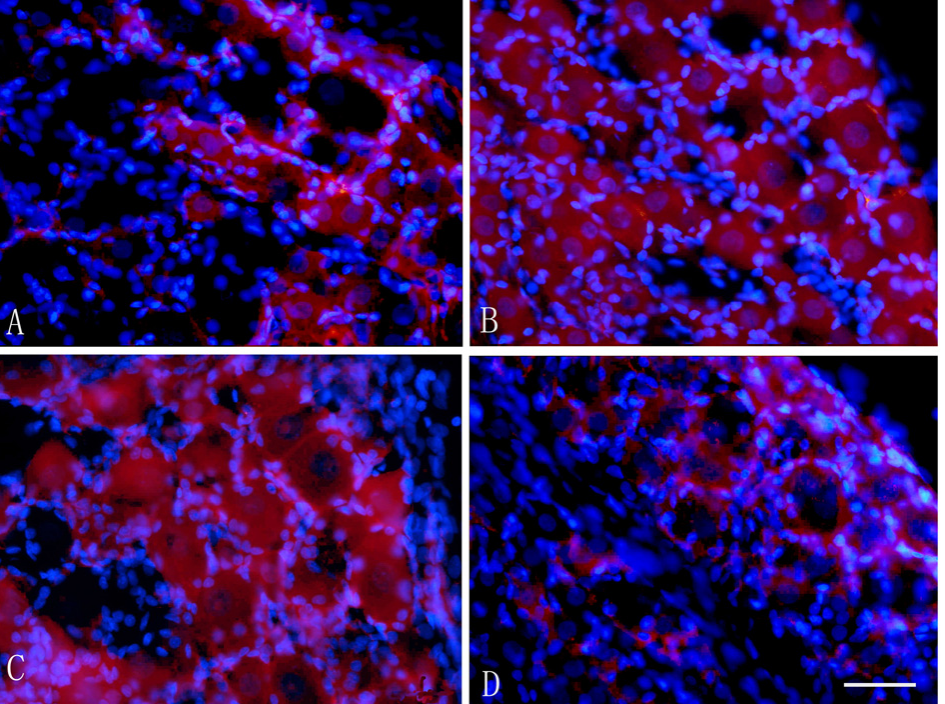
**Increased BDNF expression in the DRG after hind-paw incision**. Representative photomicrographs showing immunostaining of BDNF (red) in the ipsilateral DRG after hind-paw incision (Figure 3B-D) or the control (Figure 3A). The nuclei were stained by Hoechest (blue). Increased BDNF expressing neurons were observed at 1 hour after incision (Figure 3B) and sustained 1 day after incision (Figure 3C). The expression of BDNF at 3 days after incision (Figure 3D) went back to the basal level. Bar = 40 μm.

Similar to the expression pattern in the spinal cord in response to the spinal cord, the BDNF-expressing cells (red) in the DRG were increased 117% at 1 hour (Figure 3B) and 163% at 6 hour (Figure 3C) in the ipsilateral DRG following incision as compared with those in the contralateral DRG (Figure 3A)(p < 0.05, Table 2). The increased BDNF expressing neurons sustained more than 1 day following incision (Table 2). At 3 days after surgery, the number of BDNF-IR cells in the DRG (Figure 3D) in the ipsilateral side of incision went back to the basal level (Table 2). Quantitative analysis showed that the increased BDNF-IR was mainly expressed in the large-sized neurons in ipsilateral DRG, However, the percentage of BDNF-IR in the small-sized neurons was decreased dramatically after surgical incision (Table 3). This suggests that surgical incision induce the shift of BDNF expression from small-sized neurons to large-sized neurons in DRG.

**Table 2:** Percentage of BDNF-IR neurons in the DRG (L4–L5) after hind-paw incision

Percentage of BDNF-IR neurons	L4-L5 DRG				
	
	Sham	1h	6h	1d	3d
Contra	12.8 ± 1.8	14.5 ± 1.4	13.4 ± 1.6	14.6 ± 2.4	14.3 ± 2.1
Ipsi	13.4 ± 2.1	31.5 ± 3.7*^#^	35.3 ± 4.4*^#^	34.7 ± 3.6*^#^	16.1 ± 2.5

* p < 0.05 vs contralateral side, ^#^p < 0.05 vs sham-operated group

**Table 3:** Percentage of different size BDNF-IR neurons in total BDNF-IR neurons in DRG at 6 hrs after hind-paw incision

	BDNF-IR neurons size		
	small	medium	large
	
Contra	0.63 ± 0.12	0.30 ± 0.08	0.07 ± 0.01
Ipsi	0.35 ± 0.10*^#^	0.36 ± 009	0.28 ± 0.11*^#^
Sham	0.66 ± 0.13	0.26 ± 0.07	0.08 ± 0.03

*p < 0.05 vs contralateral side, ^#^p < 0.05 vs sham-operated group.

DRG neuron size: small: < 20 μm, medium: 20–40 μm; large: > 40 μm

**Figure 4:**
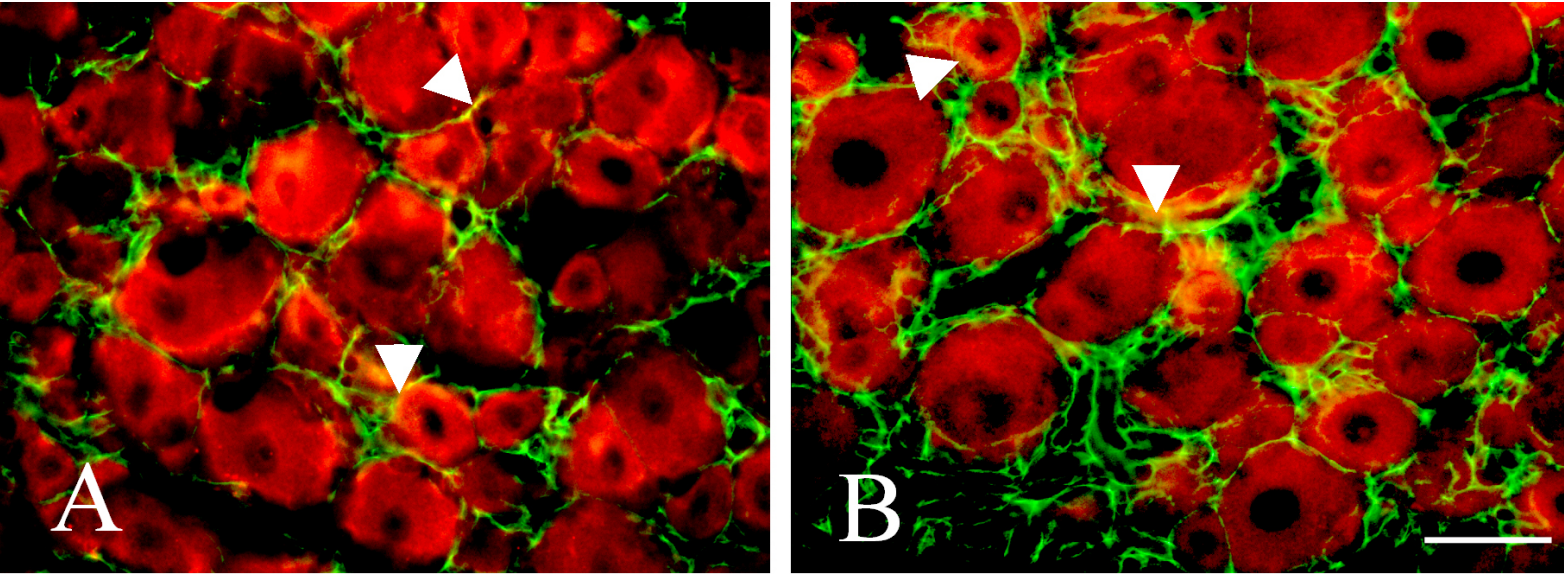
**Localization of BDNF in the DRG after hind-paw incision**. Representative double immunofluorescence labeling of BDNF (red) with S-100 (Green) (Figure 4A) and GFAP (green) (Figure 4B) in the ipsilateral DRG after hind-paw incision for 6 hours; arrow indicate double labeling. Bar = 50 μm.

To further determine whether the upregulation of BDNF was also from glial cells, double labeling of BDNF either with S-100 or GFAP, both of which are the markers for satellite cells and Schwann cells in DRG, were performed. As shown in Figure 4, a few BDNF-IR cells were also co-localized with satellite cells surrounding the neurons indicating that surgical incision activates glia cells in DRG to release BDNF.

### Involvement of afferent nerve transmission in the incision-mediated BDNF upregulation

**Figure 5:**
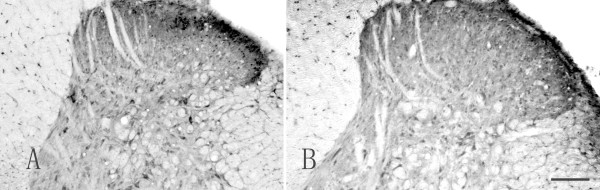
**Incision-induced BDNF upregulation through nerve transmission**. Representative photomicrographs of BDNF-IR in the ipsilateral lumbar spinal cord pretreated by saline (Figure 5A) or lidocaine (Figure 5B) sciatic nerve block followed by hind-paw incision. Bar = 150 μm.

To further investigate the role of nerve transmission in the increased BDNF after hind-paw incision, sciatic nerve block was performed by lidocaine injection just before incision. Successful block was confirmed by the nociceptive testing. Blocking the sensory impulses by lidocaine dramatically reduced BDNF protein level in spinal cord (Figure 5A and 5B) and DRG (Data not shown) at 1 h after incision at the ipsilateral side of incision (p < 0.05, lidocaine block vs saline injection, Table 4)

**Table 4:** Effect of sciatic nerve block on BDNF-IR expression at 1 h after incision

	OD of BDNF-IR in the dorsal horn			Percentage of BDNF-IR neurons		
	
	Sham	Saline	Lidocaine	Sham	Saline	Lidocaine
Contra	0.19 ± 0.03	0.25 ± 0.04	0.24 ± 0.05	12.8 ± 1.8	14.3 ± 1.9	14.2 ± 1.5
Ipsi	0.20 ± 0.04	0.46 ± 0.09*^#^	0.18 ± 0.04	13.4 ± 2.1	33.2 ± 4.6*^#^	13.6 ± 0.8

*p < 0.05 vs contralateral side, ^#^p < 0.05 vs sham-operated group, † p < 0.05 vs saline injection

### Neutralization of the spinal BDNF attenuated incision-induced allodynia

**Figure 6:**
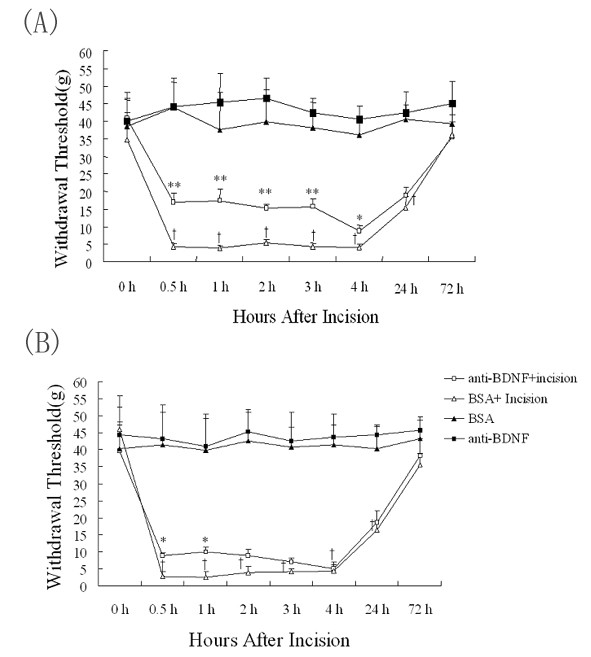
**Sequestering spinal BDNF attenuates incision-induced allodynia**. Effect of anti-BDNF antibody with IT injection (Figure 6A) or IP administration (Figure 6B) on mechanical withdrawal thresholds (g) after hind-paw incision. Single anti-BDNF antibody IT or IP injection or the saline treatment was performed 1 hour before hind-paw incision. Six to eight rats were used in each group. A, IT injection of anti-BDNF antibody dramatically inhibited the allodynia after hind-paw incision (p < 0.01, two-way repeated measure ANOVA). B, Significant difference was present between IP administration of anti-BDNF antibody (10 mg/kg) and saline treatment. *p < 0.05, group with anti-BDNF IP administration vs control by saline injection; **p < 0.01, group with anti-BDNF IT treatment vs the control by saline injection; † p < 0.01, groups after hind-paw incision vs sham-operated control.

The upregulation of BDNF in the DRG and spinal cord following incision suggests that BDNF may be involved in the incisional pain. Mechanical allodynia is the typical feature of human surgical wounds as well as the rat model of incisional pain in which allodynia sustained more than 1 day. To explore the role of BDNF in the development of mechanical allodynia induced by surgical incision, IP administration or IT injection of anti-BNDF antibody were performed before surgical incision. As shown in Figure 6A, pretreatment of anti-BDNF antibody by IT injection greatly inhibited the mechanical allodynia induced by incision. In a sharp contrast, IP injection of anti-BDNF antibody had only marginal effect on the mechanical allodynia induced by incision (Figure 6B).

## Discussion

There are several interesting findings in the present study that sought to identify the role of BDNF in the incisional pain. First, hind-paw incision induced transient activation of BDNF expression, mainly localized in the nerve terminals and neurons in the lumbar DRG and spinal cord; Second, the increased BDNF expression was changed to large-sized neurons from the small-sized neurons in the DRG in response to surgical incision; Third, sciatic nerve block by local anesthetic can prevent the upregulated BDNF in the DRG and spinal cord; Finally, IT administration, but not IP administration of anti-BDNF antibody dramatically reduced the mechanical allodynia developed by the incision.

Like inflammatory pain and neuropathic pain, incisional pain also induced the upregulation of BDNF in the DRG and spinal cord in the present study. However, the expression pattern of BDNF in the incisional pain was distinct from those in the inflammatory pain and neuropathic pain. In neuropathic pain, the increased BDNF in the spinal cord was mainly expressed in the activated microglia [18]. On the other hand, in the CFA-induced inflammatory pain model, BDNF expression was considerably increased in the small- and median-sized neurons in the DRG and the axon fibers in the dorsal horns [19]. In the present study, surgical incision induced the activation of BDNF in the large-sized neurons in DRG and the axon fibers and neurons in the dorsal horns indicating a unique BDNF expression in response to surgical incision. The distinct expression pattern of BDNF in these three types of pain strongly indicated that there might be different roles of BDNF in these three types of pain and the incisional pain has a unique mechanism as compared with the other two types of pathological pain. More interestingly, the elevated BDNF level in DRG was also localized in the large-sized neurons although BDNF is normally expressed in the small- and medium-sized neurons. Given that the central fibers of large-sized neurons in DRG mainly project to the deep layers of spinal cord which are closely related to mechanical allodynia, the phenotypic changes of the elevated BDNF in the present study strongly suggest that the activated BDNF is involved in the development of mechanical allodynia after surgical incision.

In the present study, IT injection of BDNF antibody attenuated the mechanical allodynia after incision strongly suggesting that BDNF was implicated in the incision-evoked mechanical allodynia. However, it remains elusive the underlying mechanism that BDNF contributes to the incision-evoked pain hypersensitivity. Ample evidence demonstrated that incision-evoked pain hypersensitivity was mainly mediated by AMPA/kainite-dependent mechanisms, but not NMDA receptor-dependent mechanism. This is because the AMPA antagonist but not NMDA receptor antagonist can reverse the incision-evoked pain hypersensitivity [11-13]. However, the data from mice and rat in vitro spinal cord preparation showed that BDNF led to hyperalgesia via an NMDA-mediated mechanism after peripheral inflammation [3,20]. Together with previous studies, the present study suggests that BDNF modulate the incision-evoked pain hypersensitivity through non-NMDA receptors such as TrkB or ERK. In this regard, compelling evidence showed that intraspinal injection of BDNF could phosphoryate ERK in the spinal cords, which subsequently activate the downstream signals including the pain-related neurotransmitters [21] or the related inflammatory mediator, IL-1β[17].

It has been reported that NGF contributed to the neuropathic pain and inflammatory pain, and was also associated with incision-evoked pain hypersensitivity [14]. The increase of NGF in the subcutaneous tissue after hind-paw incision was coincided with the pain-related behavior, and systemic anti-NGF treatment could reverse the incision-evoked guarding pain, but not mechanical allodynia [14]. In spite of the fact that activation of BDNF was one way that NGF contributed to inflammatory pain [2,22], BDNF might play the differential roles in the incision-evoked pain hypersensitivity as compared with NGF. In the present study, the fact that IT injection, but not systemic treatment of anti-BDNF antibody reduced the incision-evoked mechanical allodynia strongly indicated that BDNF might be involved in the incision-evoked mechanical allodynia whereas NGF be associated with spontaneous-like pain after incision.

Considerable evidence shows that inflammatory mediators in the spinal cord are activated segmentally in response to surgical incision. It has been reported that bilateral hind paw incision induce mild increase of cyclooxygenas 2 (COX-2) in the lumbar spinal cord but not cervical segments [23,24]. Our recent study also showed that surgical incision induced the segmental upregulation of IL-1β in the spinal cord [17]. In the present study, incision also induced the increase of BDNF level in the ipsilateral lumbar segments but not that in the contralateral side or thoracic spinal cord suggesting the expression pattern of BDNF level in the spinal cord was similar to that of COX-2 or the cytokines. However, unlike the activated inflammatory mediators which were not affected by the sciatic nerve block [23,24], the increased BDNF in the DRG or spinal cord could be totally prevented by the sciatic nerve block. These strongly indicate that the underlying mechanisms that incision-mediated the activation of inflammatory mediators and incision-mediated BDNF upregulation in the spinal cord are different. The rapid increase of BDNF in the DRG and spinal cord also rules out the possibility of the retrogradely transport of BDNF from the local injured tissue [25]. The fact that pretreatment of lidocaine completely prevented the upregulation of BDNF after incision indicated that the increased BDNF is through by nerve afferent transmission. In this regard, considerable evidence shows that neuronal depolarization induced by KCL or electrical stimulation of C fibers can induce the sustained increase of BDNF expression in cerebellar granule or sensory neurons [26-28]. In the present study, the increased BDNF is likely from the de nono synthesis in the large-sized neurons in DRG and novel induction in the spinal cord due to the activated burst of firing from the sensitized nerve endings in the injured tissue. The upregulated BNDF, in turn, may activate downstream signaling and contribute to the development of mechanical allodynia following incision.

In the present study, the IT injection but not IP injection of anti-BDNF antibody substantially inhibited incision-evoked mechanical allodynia suggesting that the spinal BDNF may play more important role for the incision-evoked pain hypersensitivity. Thus, blocking the upregulated spinal BDNF may be a novel potential therapeutic approach to treat post-operative pain. It has been well known that alleviation of the post-operative pain, on the other hand, can reduce the anxiety and provide subjective comfort, and should also blunt the autonomic and somatic reflex response and thus restore organ functions and enable mobilization and food intake, thereby helping to improve the outcome. Multimodal analgesia has been suggested to treat post-operative pain in which combination of different modalities, working at different pain mechanisms and reduce the side effect. In this regard, the opioids, paracetamol, NSAIDs, and COX-2 inhibitors are widely used as the pharmaceutical interventions to treat pain [29]. In the present study, the mechanism of incision-mediated BDNF may be different from that of upregulated COX-2 or cytokine induced by incision. It still remains to be determined whether BDNF antagonist (or inhibitor) can interact with other analgesia or not. However, the fact that neutralizing the increased BDNF induced by incision can reduce the mechanical allodynia following incision strongly suggest that inhibiting BDNF may be a new approach in the multimodal analgesia to treat post-operative pain.

## Conclusion

The present study demonstrated that incision induced the segmental upregulation of BDNF in the DRGs and spinal cords through afferent nerve transmission. The expression pattern of BDNF was distinct from those in the inflammatory pain and neuropathic pain suggesting that BDNF have a differential role in the incision-evoked pain hypersensitivity with neuropathic pain and inflammatory pain. IT injection of anti-BDNF antibody, but not systemic administration of anti-BDNF antibody dramatically reduced the developed mechanical allodynia following incision indicating that spinal BDNF play an important role in the incision-evoked mechanical allodynia. Thus, inhibition of the spinal BDNF may be a new approach to treat post-operative pain.

## Materials and Methods

### Animals

The study was carried out on male Wistar rats (150–250 g) obtained from Central South University Animal Services (Changsha, China). The experimental protocol was approved by the Animal Care and Use Committee of Central South University and conformed to the National Institutes of Health Guide for the Care and Use of Laboratory Animals. All efforts were made to minimize the number of rats used and their suffering.

### Surgical preparation and groups

Animals were briefly anesthetized with 1.5% isoflurane in oxygen, and an incision was made in one hind paw as described previously [10]. Using sterile technique, a 1-cm long longitudinal incision was made into the plantar skin with a no. 11 scalpel blade, starting 0.5 cm from the edge of the heel. The plantaris muscle was elevated and incised longitudinally (0.5 cm) with the blade. The skin was closed with 4-0 nylon sutures using an everted mattress pattern, and a topical triple antibiotic ointment was applied to the plantar hind paw. For immunohistochemistry and double labeling immunofluorescence, the experimental rats were randomly divided and sacrificed by 1 hour, 6 hours, 1 day and 3 days immediately after nociceptive testing. In an independent group, the rats were implanted with IT catheter for drug administration. In another independent group, the experimental rats were performed for nerve block experiment.

### Nociceptive Testing

Mechanical allodynia was assayed using nylon von Frey filaments according to the "up-down" algorithm described by Chaplan *et al *[30]. The nociceptive testing was performed by two authors who were blind to the experimental treatment. In these experiments, rats were placed on wire mesh platforms in clear cylindrical plastic enclosures of 10 cm diameter. After 20 min of acclimation, fibers of sequentially increasing stiffness were applied to the center of the plantar surface of the right hind paw between the first set of foot pads and left in place 5 s. For incised animals, the fibers were placed directly on the wound edge. Withdrawal of the hind paw from the fiber was scored as a response. When no response was obtained, the next stiffer fiber in the series was applied to the same paw; if a response was obtained, a less stiff fiber was next applied. Testing proceeded in this manner until four fibers had been applied after the first one causing a withdrawal response allowing the estimation of the mechanical withdrawal threshold.

### Intrathecal Catheterization of Rats

Isoflurane-anesthetized rats were implanted with an IT catheter modified from a method previously described [31]. After a 6d recovery period, all animals except those appeared to have sensory or motor abnormalities were used for experiments. For IT administration, 10 μl of rabbit polyclonal anti-BDNF antibody or 10 μl of isotonic saline was injected through the catheter. In an independent group, 10 mg/kg anti-BDNF antibody dissolved in the saline were performed with intraperitoneal (IP) administration. The dose of 10 mg/kg was based on our preliminary study in which three different doses of anti-BDNF antibody (2.5 mg/kg, 5 mg/kg and 10 mg/kg) were tested and no statistical significance trend was observed.

### Immunohistochemistry and double labeling immunofluorescence

Thoracic (T1–T2) and lumbar segments (L4–L5) of the spinal cord and DRGs from the control and experimental rats were fixed for 4 hours with 2% paraformaldehyde after perfusion and cryoprotected by immersion in 20% sucrose in phosphate buffer (pH 7.4) overnight. Transverse sections of the spinal cord were cut at 30 μm using a cryostat and mounted on 3-aminopropyl triethoxy-silane-coated slides, rabbit anti-BDNF antibody (dilution 1:200; Santa Cruz Biotechnology, USA) were incubated at room temperature overnight. The secondary reagents used for localization were biotinylated goat anti-rabbit IgG and ABC kit (Vector Laboratories, USA). The peroxidase reaction was visualized using 3,3'-diaminobenzidine tetrahydrochloride (DAB, Sigma, USA) as a peroxidase substrate.

Types of cells expressing BDNF was identified using double labeling. The first primary antibodies were polyclonal rabbit anti-BDNF (dilutions 1:100), and the second primary antibodies were either mouse anti-OX-42 (for microglia; dilution 1:2000, Chemicon, USA), anti-GFAP (for astrocytes in spinal cord and satellite cells in DRG; dilution 1:2000, Chemicon, USA), anti-NeuN (for neurons; dilution 1:1000, Chemicon, USA) or anti-S-100 (for satellite and Schwann cells, dilution 1:500, Chemicon, USA). The respective first and second primary antibodies were applied to the same sections simultaneously. After the overnight incubation, sections were then placed in 5 μg/ml Alexa Fluor 488 goat anti-mouse and 594 goat anti-rabbit IgG_1 _conjugate (Molecular Probes, Eugene, OR, USA) for 1 h in the dark at room temperature. After further washes, the preparations were mounted in Vectashield mounting medium (Vector Laboratories, USA). Negative controls were performed routinely by incubating the sections without either the primary or secondary antibodies.

### Nerve Block Experiment

Ten minutes before foot incision, animals were injected ipsilaterally with local anesthetic using a 25-gauge needle placed into the sciatic notch. Animals were injected with lidocaine hydrochloride 1% or normal saline, applying 0.5 ml into each sciatic notch. Sensory nerve block was confirmed by von Frey filament testing of mechanical hyperalgesia, which demonstrated an elevation of withdrawal force threshold to 144 g or greater in rats receiving local anesthetic block of the sciatic nerve (*vs*. a hypersensitive 20 g threshold in control incision animals).

### Image analysis

HPIAS-1000 image analysis software (Tongji Qingping Company, PRC) was used for the image analysis. Samples were viewed under a light microscopy (Motic). Images were captured using a digital camera (Nikon) attached to the microscope. Capture parameters were initially established and kept unmodified for all images. Five sections from each specimen and five visual fields for each section were randomly quantified. In order to define the density of BDNF immunoreactivity (-IR), we evaluated the relative optic density (OD) of positive staining. For analysis of BDNF-IR cells, cells were confirmed positive-labeled if their density level was over five times background levels [32]. Under 200 × magnification, the OD value of BDNF-IR products in the spinal cord and BDNF-IR neurons in the DRG were measured, and then the mean OD and the percentage of the positive labeling cells were calculated as described by a previous study [33]. Profiles of the DRG neurons were divided, based on size, into small (< 20 μm), medium-sized (20–40 μm), and large (> 40 μm). All measurements were performed by an author who was blind with respect to treatments.

### Statistical Analysis

Statistics analysis was performed using software SPSS 13.0 (SPSS Inc). The OD of the BDNF-IR products and percentage of BDNF-IR neurons were expressed as mean ± SD. Analysis was accomplished using a one-way analysis of variance (ANOVA) followed by *post hoc *Dunnett testing for the BDNF expression or a two-way analysis of variance followed by Bonferroni testing for nociceptive testing. A value of p < 0.05 was considered as significant difference.

## Competing interests

The authors declare that they have no competing interests.

## Authors' contributions

LCQ participated in the experiments and performed data analysis and wrote the draft of the manuscript. XJM and LD performed the animal models, IT catherarization and nociceptive testing. LCQ and ZJY performed the immunohistochemistry and the analysis of the OD. DRP conceptualized the hypothesis, designed and supervised the experiments, directed the data analysis, and revised the manuscript. All the authors have read the manuscript and agreed to submit to the journal.
